# Exploring the Burden on Patients Living With and Receiving Treatment for Immune Thrombocytopenia (ITP): Patient and Physician Perceptions From the ITP World Impact Survey (I‐WISh) 2.0

**DOI:** 10.1002/ajh.70379

**Published:** 2026-06-03

**Authors:** Nichola Cooper, James Bussel, Waleed Ghanima, Drew Provan, Yoshiaki Tomiyama, Ming Hou, Donald M. Arnold, Cristina Santoro, Francesco Zaja, Barbara Lovrencic, Mervyn Morgan, Michal Winograd, Jennifer DiRaimo, Danielle Boyle, Olivera Rajkovic‐Hooley, Meritxell Vendranas, Susan Frade, Caroline Kruse

**Affiliations:** ^1^ Imperial College Hammersmith Hospital London UK; ^2^ Weill Cornell Medicine New York New York USA; ^3^ Østfold Hospital Trust Kalnes Norway; ^4^ Institute of Clinical Medicine University of Oslo Oslo Norway; ^5^ Barts and the School of Medicine and Dentistry London UK; ^6^ Osaka University Hospital Osaka Japan; ^7^ Qilu Hospital of Shandong University Shandong University Jinan China; ^8^ Michael G. DeGroote Centre for Transfusion Research McMaster University Hamilton Ontario Canada; ^9^ Sapienza University Rome Italy; ^10^ DSM University of Trieste Trieste Italy; ^11^ Department of Hematology Azienda Sanitaria Universitaria Giuliano Isontina Trieste Italy; ^12^ AIPIT Aps—Italian Association of Immune Thrombocytopenic Purpura Caprino Veronese Italy; ^13^ ITP Support Association Bolnhurst UK; ^14^ ITP Israel, the Israeli Association of ITP Ramat Hasharon Israel; ^15^ GenomiCare Toronto Ontario Canada; ^16^ ITP Australia and New Zealand Cairns Queensland Australia; ^17^ Adelphi Real World Bollington UK; ^18^ Novartis Farmacéutica Barcelona Spain; ^19^ Novartis Pharma AG Basel Switzerland; ^20^ Platelet Disorder Support Association Cleveland Ohio USA

**Keywords:** disease burden, immune thrombocytopenia, platelets—disorders of platelets, quality of life, treatment impact

## Abstract

Limited data exist on how patients and physicians perceive immune thrombocytopenia (ITP) symptoms and treatment‐related burden. I‐WISh (ITP World Impact Survey) 2.0 surveyed 1018 patients and 431 physicians in 15 countries to characterize the impact of ITP and its treatments on patients. Approximately one‐third of patients reported that ITP had a high impact on daily activities and family/social life, and 54% reported that it had a high impact on emotional wellbeing. Patients and physicians generally aligned on symptoms and treatment goals, but more patients (48%) than physicians (29%) reported fatigue as common and problematic. Ninety‐seven percent of patients reported that they had received treatment for ITP, most commonly corticosteroids. Most patients and 54% of physicians were satisfied with available treatments, although patients reported treatment‐related burdens. Twenty‐eight percent confirmed they would have chosen a different treatment. Two‐thirds of patients were in a stable, sustained remission, and most had never paused treatment (67%). Nevertheless, two‐thirds of patients preferred limiting time on treatment and/or not being on lifelong treatment. I‐WISh 2.0 emphasizes the extensive disease and treatment burden faced by patients living with ITP, especially the impact of fatigue. It also reminds us that shared decision‐making is needed to ensure treatment goals are aligned with patient expectations, including health‐related quality of life.

## 1 Introduction

Immune thrombocytopenia (ITP) is an autoimmune disorder characterized by reduced platelet counts and increased bleeding [1, 2], with an estimated incidence of 1.9–6.4/100 000 children/year and 2–4/100 000 adults/year [3, 4]. ITP signs and symptoms include bleeding (from petechiae and purpura to more serious but rare gastrointestinal bleeding and intracranial hemorrhage), fatigue and psychological symptoms (e.g., anxiety and depression), as well as cognitive impairment and possibly also headache [1, 2, 5, 6, 7, 8]. These reflect the multifaceted impact of ITP, ranging from direct effects, i.e., bleeding, to broader, more extensive, complex and systemic effects on the totality of patient health‐related quality of life (HRQoL) [2, 3, 4, 5].

Approved treatments for ITP include corticosteroids, intravenous immunoglobulin (IVIg), thrombopoietin receptor agonists (TPO‐RAs), fostamatinib, and splenectomy. Rituximab and immunosuppressive agents (e.g., mycophenolate mofetil) are widely used off‐label [9, 10]. The complex pathophysiology of ITP means some patients will not respond to initial therapies and a proportion will not respond to subsequent treatments [9, 10]. Furthermore, responding patients may not stably maintain their response and/or develop toxicities leading to treatment change [9, 10]. Patients maintaining responses on treatment may trial discontinuing them; however, these responses are often not sustained [9, 10]. Splenectomy is rarely used currently because it does not always work and carries a lifelong risk of serious complications [9, 10]. Finally, treatments that successfully improve platelet counts may worsen patients' HRQoL [9, 10].

I‐WISh (ITP World Impact Survey) 1.0 was a cross‐sectional survey completed between December 2017 and August 2018 by 1507 patients with ITP and 472 physicians treating ITP [11, 12]. It confirmed that ITP had substantial adverse impacts on patient HRQoL, including social, work and school lives, and emotional wellbeing while extending and better defining the extent of these problems [11]. While both patients and physicians rated sustained remission and bleeding risk reduction as the most important treatment attributes, improving HRQoL was also a very important goal [12].

I‐WISh 2.0 built on findings from I‐WISh 1.0 to better characterize ITP disease and diagnostic burden, treatment perceptions, and management. By expanding to new countries and exploring fatigue, mental health, and emotional impact more deeply, this study identified unmet needs and improved understanding of ITP worldwide. We report the impact of living with ITP on patients, investigate perceptions of ITP treatments and their impact on HRQoL and emotional wellbeing, as well as explore treatment satisfaction, goals, and sustained responses after treatment discontinuation.

## 2 Methods

I‐WISh 2.0 was a cross‐sectional survey conducted between February and July 2022 of patients with ITP and physicians who treat ITP from 15 countries (Australia, China, Egypt, France, Germany, India, Israel, Italy, Japan, Norway, South Korea, Spain, Taiwan, UK, and USA). Patients and physicians who had participated in I‐WISh 1.0 could complete I‐WISh 2.0 if they met the selection criteria (same criteria for both surveys; Supporting Information Methods). Patients > 18 years of age with ITP were recruited by physicians or patient associations. Hematologists or hemato‐oncologists managing ≥ 3 patients with ITP in the past 12 months were invited by fieldwork partners. Participants received an anonymized survey link (a printed version was used in Japan) and fully de‐identified respondent information was aggregated.

Patient and physician surveys were developed with repeated rounds of editing by a steering committee comprising expert clinicians and patient advocates representing patient associations across several countries, Adelphi Real World (ARW), and Novartis Pharma AG. Data collection and analyses were managed by ARW. Surveys were developed in English, then translated (Supporting Information Methods). Patient and physician surveys each comprised eight sections: demographics; symptoms; impact of fatigue; impact on daily life; disease management and treatment; emotional impact; impact on pregnancy (optional); and impact of COVID‐19 on ITP (optional; Supporting Information Methods). Respondents did not have to answer all questions, and certain questions only became available in response to specific answers; therefore, the number of respondents for each question could vary. The questions in the patient survey were not prescreened by patients, but data collection was initiated in the UK and USA and results reviewed to ensure correct functioning of the programmed surveys before roll‐out of the survey to the full sample. Survey details are outlined in the Supporting Information Methods.

Patient and physician surveys were analyzed separately. Data were summarized using descriptive statistics (Supporting Information Methods). There were no prespecified hypotheses associated with these exploratory surveys and, thus, no control group. For outcomes measured on a 7‐point Likert scale, scores of 5–7 were used to denote severe symptoms (assessed on a scale of 0 [*not severe at all*] to 7 [*worst imaginable*]), high impact (0 [*not at all*] to 7 [*a great deal*]), importance (0 [*not important at all*] to 7 [*extremely important*]), or agreement (0 [*strongly disagree*] to 7 [*strongly agree*]). Platelet counts at diagnosis were compared with those at the most recently conducted test and the change in symptoms from diagnosis to survey completion was assessed. In addition, emotional wellbeing was evaluated according to percentage change in symptoms from diagnosis (0% to < 25% increase, 25%–50% increase, > 50% increase, no change, 0% to < 25% decrease, 25%–50% decrease, > 50% decrease), and emotional wellbeing according to fatigue: yes, no (Supporting Information Methods). Subgroup analyses according to age, disease phase, platelet count, and reporting of fatigue were conducted for key outcome measures (see Supporting Information Methods).

## 3 Results

One thousand and eighteen patients and 431 physicians completed I‐WISh 2.0; China, USA, and UK recruited the most participants (Table 1). Because of the recruitment methods, the percentage of completed surveys could not be obtained. Only 4% of patients (*n*/*N* = 43/1018) and 16% of physicians (70/431) had completed I‐WISh 1.0. Median patient age was 46 years, 67% were female, and median time from ITP diagnosis was 50 months. At diagnosis, 87% of patients (736/850) had platelet counts < 50 × 10^9^/L, whereas for 70% (598/850) their most recent platelet count was ≥ 50 × 10^9^/L (Table S1). For physicians, the median number of patients with ITP under their care at survey completion was 20, and 75% had treated ITP for at least 11 years.

**TABLE 1:** Demographics of patients and physicians who completed I‐WISh 2.0.

	*N*	%^a^
**Patient demographics**		
*Patients who completed I‐WISh 2.0*	1018	—
Asia	406	40
China	150	15
India	60	6
South Korea	60	6
Japan	57	6
Israel	40	4
Taiwan	39	4
Europe	331	33
UK	99	10
France	72	7
Italy	70	7
Germany	54	5
Norway	29	3
Spain	7	< 1
North America	145	14
USA	145	14
Australia	76	8
Africa	60	6
Egypt	60	6
*Route of recruitment*		
Physician	693	68
Patient association	325	32
*Sex*		
Female	686	67
Male	327	32
Prefer not to say	5	< 1
*Age (years)*		
18–40	380	37
41–60	415	41
61 or older	223	22
*Median age, years (IQR):* 46 (35–59)		
*Median time since ITP diagnosis, years (IQR):* 4.2 (2–10)		
*Phase of disease*		
Newly diagnosed	22	2
Persistent	127	12
Chronic	869	85
*Stable platelet count*		
Yes	562	55
No	327	32
Don't know	129	13
*Platelet count at most recent test*		
< 10 × 10^9^/L	36	4
10–19 × 10^9^/L	41	4
20–29 × 10^9^/L	70	7
30–49 × 10^9^/L	139	14
50–99 × 10^9^/L	269	27
100–199 × 10^9^/L	263	26
≥ 200 × 10^9^/L	129	13
Unknown	61	6
*Treatment being used at the time of survey completion*		
TPO‐RAs	244	26
Corticosteroids	232	24
Following ‘watch and wait’	165	17
Immunosuppressants (e.g., cyclosporine, azathioprine, cyclophosphamide, and mycophenolate mofetil)	100	10
Intravenous immunoglobulins	75	8
Platelet transfusion	64	7
Antibiotics (e.g., dapsone)	44	5
Herbal medicines/natural therapies	43	5
Androgens (e.g., danazol and testosterone)	43	5
Anti‐CD20 (e.g., rituximab, veltuzumab, and ofatumumab)	31	3
Anti‐fibrinolytic	27	3
Rho(D) immunoglobulin	19	2
Other	37	4
Can't remember treatment name	41	4
None^b^	142	15
**Physician demographics**		
*Physicians who competed I‐WISh 2.0*	431	—
Asia	195	45
China	100	23
Japan	30	7
India	20	5
South Korea	20	5
Taiwan	20	5
Israel	5	1
Europe	157	36
Italy	31	7
France	30	7
Spain	30	7
UK	30	7
Germany	25	6
Norway	11	3
North America	59	14
USA	59	14
Africa	20	5
Egypt	20	5
Australia	—	—
*Specialty*		
Hematology	295	68
Hematology and oncology	136	32
*Clinical setting*		
University/teaching hospital	257	60
Regional or community hospital	79	18
Specialist cancer center	47	11
Private hospital	29	7
Office based	10	2
Other	9	2
*Time treating patients with ITP*		
< 5 years	21	5
5–10 years	86	20
11–15 years	112	26
16–20 years	112	26
21 years or more	100	23
*Median number of patients with ITP under care at survey completion (IQR):* 20 (10–35)		

Abbreviations: I‐WISh 2.0, ITP World Impact Survey 2.0; IQR, interquartile range; ITP, immune thrombocytopenia; TPO‐RA, thrombopoietin receptor agonist.

^a^ Percentages represent the proportion of respondents per subgroup out of the total number of respondents. Percentages may not total 100% because of rounding.

^b^ Never received prescribed treatment for ITP.

### 3.1 ITP Symptoms and Severity

**FIGURE 1:**
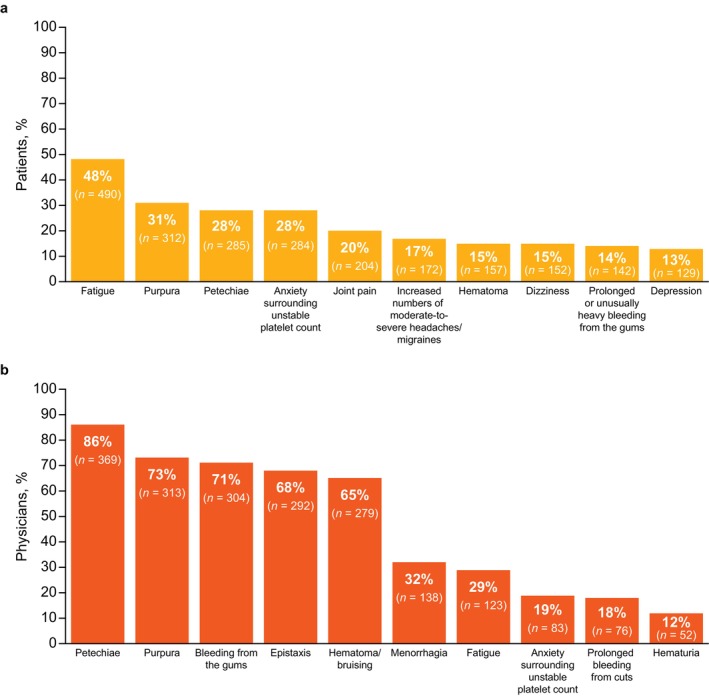
Most frequent ITP symptoms as (a) self‐reported by 1018 patients during the month prior to survey completion and (b) identified by 431 physicians as commonly mentioned by their patients (both irrespective of the stage of ITP). ITP, immune thrombocytopenia.

In the month before survey completion, the ITP symptoms reported most commonly by patients were fatigue, purpura, petechiae, and anxiety surrounding unstable platelet counts (Figure 1). Heavy menstrual bleeding was reported by 15% of women (104/686). For each symptom reported by patients, fatigue (54%, 262/487), anxiety surrounding unstable platelet counts (52%, 146/283), and depression (46%, 59/129) were most frequently described as severe. The top three symptoms patients wanted resolved were fatigue (42%, 425/1018), purpura (22%, 219/1018), and anxiety around unstable platelet counts (19%, 194/1018), with almost one‐quarter (24%, 240/980) reporting an unstable platelet count. Physicians recognized petechiae (86%, 369/431), purpura (73%, 313/431), and gum bleeding (71%, 304/431) as symptoms most frequently mentioned by their patients (irrespective of the stage of ITP), followed by epistaxis (68%, 292/431) and hematoma/bruising (65%, 279/431) (Figure 1). Twenty‐nine percent of physicians (123/431) reported fatigue as one of the five symptoms most frequently mentioned by patients.

Since diagnosis, only 19% (194/1018) of patients saw an overall decline in their health status; 55% reported an improvement (557/1018) and 26% (267/1018) reported no change. Increased platelet counts at survey completion compared with diagnosis were reported by 80% (684/850) of patients; 13% (110/850) reported no change and 7% (56/850) a decrease. Although most patients reported a decrease in the number of ITP symptoms at the time of survey completion compared with diagnosis (66%, 661/1006), 17% (175/1006) reported more symptoms and 17% (170/1006) reported no change. When assessing percentage change in symptoms from diagnosis, 12% (115/957) of patients reported a > 50% increase in symptoms (Table S2). Similar types of symptoms were reported at the time of diagnosis and during the month prior to survey completion, by both patients and physicians (Supporting Information Methods).

### 3.2 Impact of ITP on Functional Aspects of Patients' HRQoL, Including Work and Activities

**FIGURE 2:**
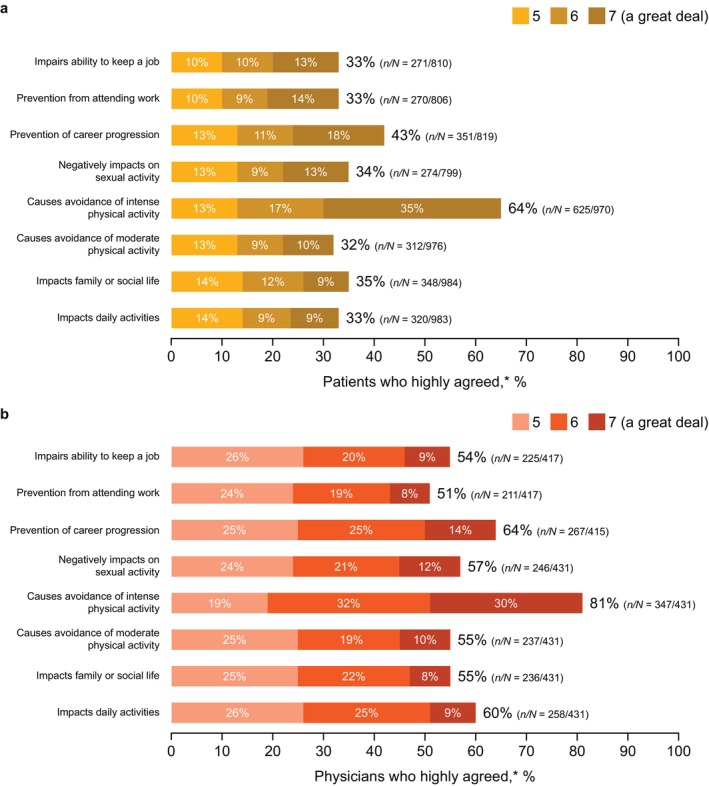
Percentage of (a) patients experiencing and (b) physicians reporting a high impact* of ITP on patients' daily lives. *n* represents the total number of participants who gave this answer and *N* denotes the total number of participants who completed the question. *Highly agreed meant scoring 5–7 on a Likert scale. ITP, immune thrombocytopenia.

Approximately one‐third of patients reported that ITP had a high impact on various aspects of their lives, including daily activities and family/social life; the proportion of physicians reporting a high impact on these aspects of patients' lives was consistently higher (Figure 2). When analyzed according to subgroups, the proportions of patients and physicians reporting a high impact on daily activities and family/social life were slightly higher (> 10%) in those with lower platelet count < 100 (vs. ≥ 100) × 10^9^/L but did not appear to be affected by disease phase (Table S3).

One‐third (32%, 312/976) of patients reported avoiding moderate physical activity and 64% (625/970) avoid intense physical activity; overall, avoidance rates were higher in subgroups reporting fatigue (vs. no fatigue), but were not affected by age, advancing disease phase, or platelet count (Table S4). Thirty percent (246/830) of patients reported a high impact of ITP on sexual activity. In support of this, 89% (385/431) of physicians reported that ITP symptoms had a high impact on their patients' quality of life (QoL), affecting multiple aspects of daily life including alertness, traveling, and maintaining physical and mental effort (Supporting Information Methods).

Patients and physicians both felt that patients' careers were affected by ITP. At work, 43% (351/819) of patients agreed that their ITP prevented their career from progressing, 33% (270/806) were prevented from attending their job, and 33% (271/810) felt that ITP impaired their ability to keep a job. Many patients (40%, 348/863) worried that they were unable to work or contribute financially, 45% (421/936) felt like a burden, and 40% (366/916) wished they were able to contribute more to society. Roughly two‐thirds of physicians (64%, 267/415) agreed that ITP prevents their patients' careers progressing. Patients also reported that ITP impacted their ability to concentrate on daily tasks, with 50% (513/1017) experiencing this “sometimes,” 19% (196/1017) “more than half the time,” and 5% (54/1017) “all the time.” Fifty‐nine percent of physicians (250/421) estimated that 50% or more patients have trouble concentrating because of their ITP. Most patients (57%, 491/855) felt that their sleep quality would be better if they did not have ITP and 58% of physicians (243/422) estimated that about 50% or more patients have poor sleep quality (Supporting Information Methods).

### 3.3 Impact of ITP on Pregnancy

Reflecting on their most recent/current pregnancy, 53% (62/118) had/expected to have a natural birth, 12% (14/118) had/expected to have a planned cesarean section because of their ITP, and 5% (6/118) had an emergency cesarean section because of their ITP. Seventeen percent (20/118) of patients had an epidural, but 11% (13/118) could not have one because their platelet count was too low. The proportions of patients who reported receiving ITP treatments that they had not received previously were 15% (18/118) during pregnancy, 8% (9/118) at delivery, and 14% (17/118) after delivery. Among patients whose pregnancy status was known (*n* = 682), 6% (43/682) chose not to pursue pregnancy because of their ITP. Most physicians (85%, 349/409) reported that they treated patients differently during pregnancy.

### 3.4 Impact of ITP on Patients' Emotional Wellbeing

**FIGURE 3:**
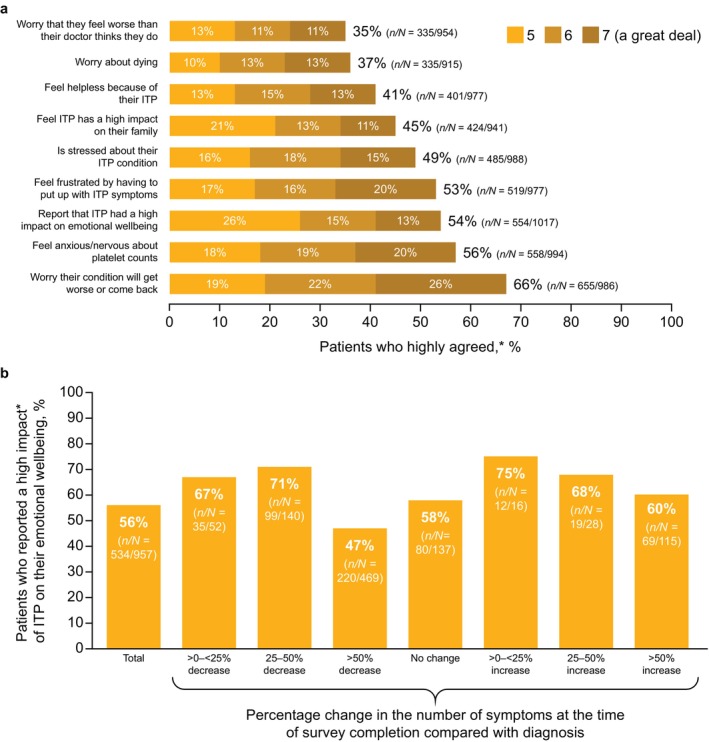
Percentage of patients with ITP experiencing a high impact* on (a) their emotional health and (b) overall emotional wellbeing according to the percentage change in the number of symptoms. *n* represents the total number of participants who gave this answer and *N* denotes the total number of participants who completed the question. *Highly agreed/high impact meant scoring 5–7 on a Likert scale. ITP, immune thrombocytopenia.

Over half of patients (54%; 554/1017) felt that ITP had a high impact on their emotional wellbeing, which included concerns about their condition worsening or coming back, feeling anxious or nervous about their platelet counts, frustration about having to deal with ITP symptoms, and stress about their ITP (Figure 3a). The proportion of patients reporting a high impact did not appear to be affected by disease stage or platelet count (Table S5). Despite reporting up to a 50% decrease in symptoms at the time of survey completion compared with diagnosis, 70% of these patients still reported a high impact on their emotional wellbeing (Figure 3b). Most physicians (77%, 331/431) agreed that ITP has a high impact on their patients' emotional wellbeing.

According to the Patient Health Questionnaire‐9 (PHQ‐9), more than half of patients (58%, 555/961) reported experiencing depression (score ≥ 5) at survey completion: mild (score 5–9) in 26% (253/961), moderate (score 10–14) in 20% (190/961), moderately severe (score 15–19) in 8% (79/961), and severe (score 20–27) in 3% (33/961). The mean PHQ‐9 score was 7.1 (mild depression; *N* = 961). Only 9% (91/1014) of patients reported receiving medication for depression/anxiety.

### 3.5 Impact of Fatigue for Patients With ITP

**FIGURE 4:**
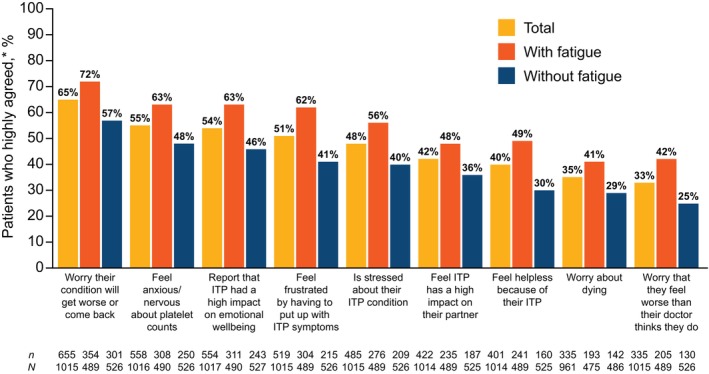
Percentage of patients experiencing high impact* on emotional wellbeing according to fatigue (with or without) experienced by patients in the month prior to survey completion. *n* represents the total number of participants who gave this answer and *N* denotes the total number of participants who completed the question. *Highly agreed meant scoring 5–7 on a Likert scale. ITP, immune thrombocytopenia.

When asked how fatigue had affected them in the 4 weeks prior to survey completion, 18% (185/1012) of patients reported they were “often” or “almost always” unable to think clearly, 22% (227/1012) were less motivated to do anything that requires thinking, 25% (255/1015) were limited in their ability to do things away from home, 25% (259/1016) were less alert, 27% (272/1015) had trouble concentrating, 35% (350/1012) were less motivated to participate in social activities, 36% (368/1015) were less able to complete tasks that required physical effort, and 37% (375/1015) had trouble maintaining physical effort for long periods of time. Most physicians agreed that fatigue had a high impact on patients' QoL and patients experiencing fatigue reported a higher impact of ITP on their emotional wellbeing than patients who did not (Figure 4).

Over half of physicians (54%, 234/431) reported an association between patients' platelet counts and fatigue, and 78% of patients who experienced fatigue (346/446) agreed. Most (81%, 359/445) patients reported experiencing fatigue when they had a low platelet count, although 34% (139/409) reported experiencing it even when their platelet count was high. Similarly, 81% (351/434) of patients reported experiencing fatigue when they had an unstable platelet count, but only 33% (137/419) reported experiencing it when their platelet count was stable. Seventy percent (302/431) of physicians agreed that patients with platelet counts of 10–19 × 10^9^/L were likely to experience fatigue, but only 11% (47/431) believed that patients with a platelet count of 100–199 × 10^9^/L were likely to experience fatigue.

### 3.6 ITP Treatment and Management

Nearly all patients (97%, 956/987) reported having been on management for ITP (pharmacological therapies, herbal/natural remedies, IVIg, platelet transfusion, anti‐D, and/or “watch and wait”) and 12% (126/1017) had a splenectomy. The most commonly reported treatments were corticosteroids (72%, 707/987), IVIg (45%, 441/987), and TPO‐RAs (37%, 367/987); one‐quarter of patients (242/987) reported having been on “watch and wait” at some point. At survey completion, most patients (85%, 811/953) reported that they were on management, most commonly TPO‐RAs and corticosteroids, with median time on most recent treatment reported as 30 weeks (*N* = 608).

The most common first‐line management physicians reported were corticosteroids (92%, 397/431), IVIg (58%, 250/431), and “watch and wait” (no therapy; 44%, 188/431); for second‐line treatment, the most common were TPO‐RAs (70%, 302/429), anti‐CD20 therapies (52%, 224/429), and IVIg (33%, 143/429).

Twenty‐four percent (136/560) of patients said they did not want to receive any treatment but their doctor insisted that they did. Physicians reported a mean of 15% of patients opting against medication for ITP. Physicians predominantly reported considering patient perceptions when making treatment decisions (73%, 313/431), although 28% (203/733) of patients affirmed they would be on a different treatment if it was their decision.

### 3.7 Patient and Physician Treatment Goals

**FIGURE 5:**
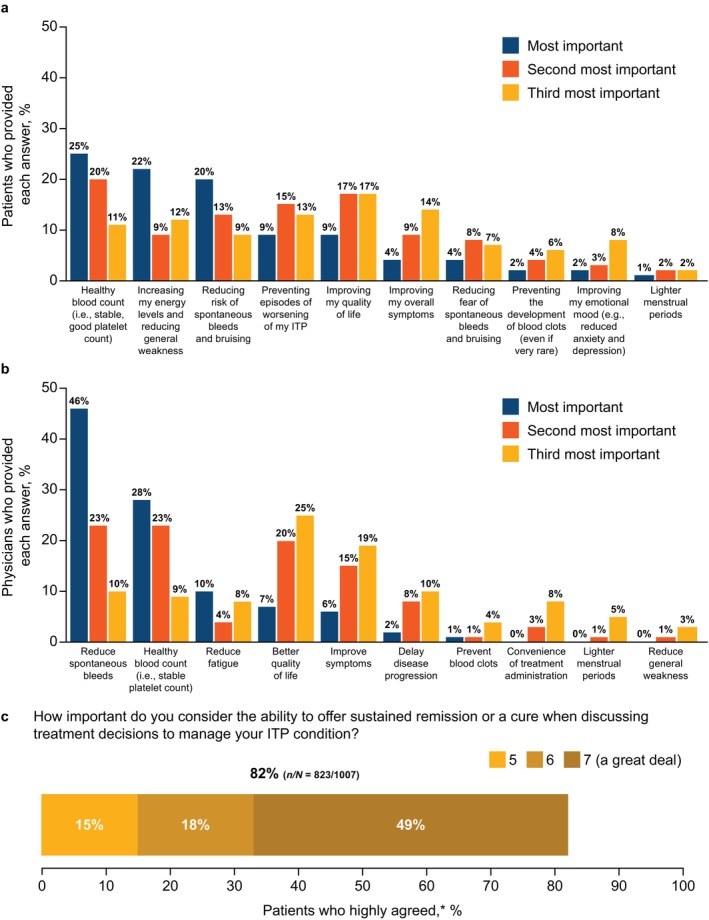
Most important treatment goals identified by (a) 1018 patients and (b) 431 physicians and (c) patient perception of importance of sustained remission during treatment decisions. *Highly agreed meant scoring 5–7 on a Likert scale. ITP, immune thrombocytopenia.

Of patients' top treatment goals, the most selected were healthy blood counts, i.e., stable platelet counts (56%, 566/1018), improving QoL (44%, 446/1018), increasing energy levels (43%, 442/1018), and reducing risk of spontaneous bleeds (42%, 427/1018). Similarly, the top three treatment goals most commonly mentioned by physicians were reducing spontaneous bleeds (79%, 340/431), healthy blood counts (60%, 258/431), and improving QoL (52%, 224/431). Figure 5 shows treatment goals ranked by patients and physicians by importance. When asked how important aspects were when discussing ITP treatment decisions, over 80% of patients considered a treatment offering a sustained remission or cure for ITP and 78% (787/1008) felt keeping side effects to a minimum were important factors.

### 3.8 Patient and Physician Treatment Satisfaction and Perception

Most patients (73%, 739/1014) agreed their current ITP management helps. Seventy percent (593/843) of patients agreed treatment was effective in treating their ITP symptoms; however, 59% (562/955) reported starting ITP treatment had a high impact on their emotional wellbeing (e.g., anxiety, stress, and mood). Most patients (73%, 656/897) were satisfied with their treatment's control of platelet levels; however, 19% (172/897) of patients were not (1–3 on a Likert scale) and 18% (155/843) disagreed that their medication effectively controlled their symptoms. Nearly half (45%, 429/950) of patients agreed that their fatigue had improved since initiating ITP treatment. Physicians agreed many ITP treatments can negatively impact fatigue (corticosteroids and other immunosuppressive treatments were reported as having the most negative impact).

**FIGURE 6:**
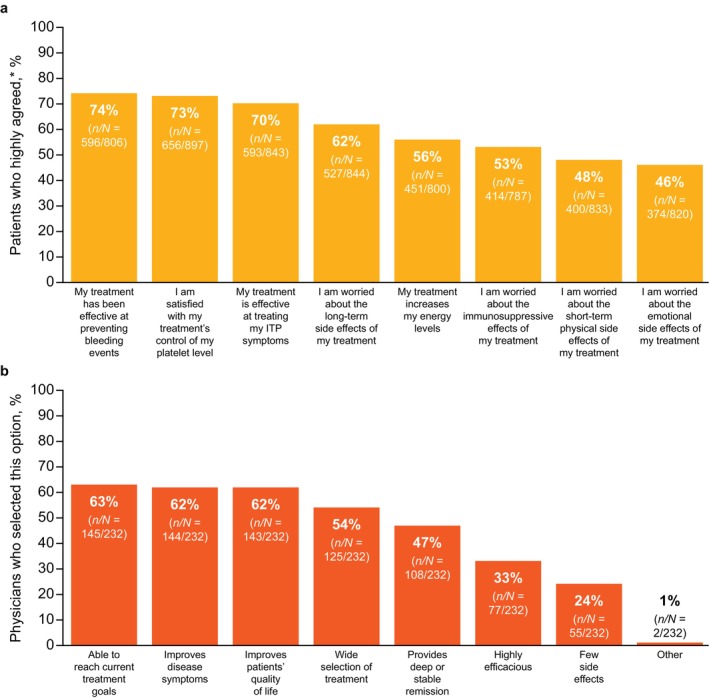
(a) Patient satisfaction with treatment and (b) reasons for satisfaction with treatment of the 232 physicians who were satisfied or very satisfied with current treatment options. *n* represents the total number of participants who gave this answer and *N* denotes the total number of participants who completed the question. *Highly agreed meant scoring 5–7 on a Likert scale. ITP, immune thrombocytopenia.

Many patients reported being worried about short‐ and long‐term side effects of treatment, including immunosuppressive and emotional effects (e.g., depression and anxiety) (Figure 6a). One‐third of patients (34%, 202/597) agreed that planning medication disrupted their lives, 37% (214/576) were bothered by planning meals, and 22% (204/939) had difficulty keeping up with daily medications. More than half of physicians (55%, 228/413) believed that patients feel a burden from dietary changes to accommodate ITP medication.

Fifty‐four percent of physicians reported satisfaction with current treatment options, with 2% (8/431) very satisfied, 52% (224/431) satisfied, 38% (162/431) neutral, 7% (31/431) dissatisfied, and 1% (6/431) very dissatisfied. Among those satisfied or very satisfied with current treatment options, the most commonly reported reasons for satisfaction included the ability to reach current treatment goals, improved disease symptoms, improved QoL, and a wide selection of treatments (Figure 6b). Less commonly reported reasons for satisfaction were the ability to provide stable remission and few side effects. The most commonly reported reasons for changing therapy were lack of efficacy (83%, 357/431), side effects (72%, 312/431), and disease worsening (70%, 302/431).

Most patients (67%, 679/1007) considered immunosuppression prevention important when discussing treatment decisions. Almost two‐thirds of physicians (62%, 268/431) agreed that a main aim is limiting immunosuppressive side effects (5–7 on a Likert scale), but only 12% (52/431) felt most strongly about this (7 on a Likert scale). Most physicians (74%, 318/431) felt that corticosteroids had a negative impact on patients' QoL.

### 3.9 Treatment Pausing Patterns and Remission

Two‐thirds of patients (67%, 680/1013) showed concern over relapsing if current treatments were stopped and approximately one‐third (37%, 52/141) of those on watch and wait were worried about the risks. When asked if they had previously paused treatment, most patients (67%, 610/915) reported never having paused treatment, 26% (241/915) reported having paused treatment, and 7% (64/915) did not know. The three most commonly reported reasons for pausing treatment were: disease was under control (42%, 101/241); platelet count was stable (25%, 60/241); and experiencing side effects from treatment (22%, 53/241). The most common reasons patients reported restarting medication after pausing were: decreasing platelet count (67%, 161/241); re‐emergence of bleeding symptoms (26%, 62/241); and the plan for treatment pause to be temporary, while acknowledging that they still required long‐term treatment (17%, 42/241).

Overall, 71% (305/431) of physicians agreed their patients were willing to pause treatment following sustained response (defined at physician discretion; Figure S1). Most physicians (70%, 300/431) reported having paused or discontinued TPO‐RAs at some point; of these, 83% (249/300) reported reducing the dose before pausing all the time, often, or sometimes.

At survey completion, only 55% (562/1016) of patients reported being in sustained remission (i.e., stable platelet count); 32% (327/1016) reported that they were not in sustained remission and 13% (127/1016) did not know. Of patients reporting sustained remission, the reported duration of clinical remission (i.e., no bleeding and not receiving treatment) was 0–3 months in 29% (164/562), 4–6 months in 22% (122/562), 7–12 months in 17% (93/562), and over 12 months in 33% (183/562).

Thirty‐eight percent (294/775) of patients did not want to take their treatment for the foreseeable future. Limiting treatment time and not being on treatment lifelong substantially affected treatment preferences for 66% (668/1009) of patients (Figure S1).

## 4 Discussion

### 4.1 ITP Impact on Patients

A key feature of our findings is the substantial burden of fatigue. It expands on findings reported in I‐WISh 1.0 [12], with 54% of patients experiencing fatigue reporting it as severe in I‐WISh 2.0. However, almost half of the patients reported fatigue had improved since starting ITP treatment. Fatigue had a pervasive negative impact on many aspects of patients' QoL, including emotional wellbeing, socializing, and sexual activity. Fatigue was more frequently reported in patients with a low versus high platelet count and in those with an unstable versus stable platelet count. Surprisingly, the experience of fatigue, rather than platelet counts per se, impacted the relatively high proportion of patients avoiding physical activity. Most physicians believed that fatigue would decrease as platelet count increased, although one‐third did not. Further research is needed to understand this potential relationship, starting with a better understanding of the complex pathophysiology of fatigue in ITP. There is mixed evidence, with one survey supporting a correlation between platelet count and bleeding with fatigue and another study reporting no correlation between fatigue and platelet count or platelet response to treatment [13, 14]. Patients considered fatigue to be more common than physicians, who, despite recognizing that fatigue has a high impact, may be more focused on bleeding and platelet counts or only recognize it when it is severe. The persistence and emotional impact of fatigue highlights a need for further clinical research, including whether and how fatigue relates to other symptoms (e.g., depression and anxiety around low or unstable platelet counts), what its physiologic basis is, and how to manage fatigue if increasing platelet counts is insufficient or difficult. Fatigue is common in other autoimmune conditions [15], suggesting it reflects chronic inflammation from immune activation [16]. Fatigue, however, is almost certainly multifactorial in ITP, so no one explanation underlies fatigue in every patient.

Almost one‐quarter of patients reported their platelet count was not stable and they listed anxiety around unstable platelet counts as one of their top three symptoms to resolve, alongside fatigue and purpura. Anxiety around unstable platelet counts was reported in 28% of patients in the month prior to survey completion, although 80% reported having an increase in platelet counts since diagnosis and almost three‐quarters described their treatment as effective. Patients reported anxiety around platelet counts at the same frequency at diagnosis and the month prior to survey completion, and physicians reported that more of their patients currently mention anxiety around platelet counts than at diagnosis. This emphasizes the need to develop a potentially curative therapy, one example being chimeric antigen receptor (CAR) T‐cell therapy, to assuage these concerns [12].

Over half of patients experienced some degree of depression at survey completion (assessed by PHQ‐9), greatly exceeding the World Health Organization's estimate of 5% among the general adult population [17] and higher than the estimate from a large cohort study of patients with primary ITP [18]. Furthermore, the proportion of patients in the survey with a PHQ‐9 score ≥ 10, a commonly used cut‐off denoting clinical depression, was 22%. This compares with 6%–13% in cross‐sectional studies in the general population [19, 20, 21, 22] and is similar to the prevalence reported in a systematic review of studies in primary ITP (16%–25% patients with PHQ‐8 ≥ 10) [23]. Even in patients reporting a decrease in symptoms of up to a 50% since diagnosis, the emotional impact of ITP remained high. Patients reporting no change or a > 50% increase in the number of symptoms since diagnosis reported a lower emotional burden than patients with a < 25% or 25%–50% decrease in the number of symptoms (Figure 3b). One explanation for this is possible adjustment to and acceptance of a sub‐optimal “normal,” a concept discussed in I‐WISh 1.0; another is that certain patients may feel better despite more symptoms because they are on less therapy. This highlights the importance of a multidisciplinary approach to patient care that not only includes nurses, but also referral to psychologists when required to better address patient concerns and foster higher, albeit achievable, expectations for HRQoL and emotional and mental health [12]. Neuropsychiatric symptoms (including anxiety and depression) are also frequent in other autoimmune disorders, and numerous pathogenic mechanisms have been suggested to account for this, with some overlap with the causes of fatigue [24].

There was consensus that ITP places significant burden on patients that affects all aspects of their life and emotional wellbeing. This is not surprising given that there are many simultaneous effects of ITP (e.g., fatigue, depression, bleeding, and familial circumstances). Furthermore, the interplay among these factors is complicated, so it is poorly understood how changing one will affect others. A patient's emotional wellbeing, ability to concentrate and energy for work, and family and social interactions can all be affected. Interestingly, lower vs. higher platelet counts (< 100 vs. ≥ 100 × 10^9^/L) were associated with a greater patient‐reported impact on daily activities and family/social life, but not emotional well‐being. Biologic and interventional studies are required to understand the optimal way to approach patients, which also may vary according to the type of problem.

Results from I‐WISh 2.0 illustrate that since I‐WISh 1.0, experienced physicians have become more aware of the multidimensional impact of ITP; for example, surprisingly more physicians than patients reported perceiving the emotional impact of ITP being high (77% of physicians vs. 54% of patients). The increased awareness of burden among physicians likely reflects this specialized group's knowledge and expertise. Alternatively, it could be another indication that patients cope with suboptimal “normal,” not recognizing that they could feel better under other circumstances (i.e., without ITP or with ITP that is improved by treatment or over time). Other possibilities include that patients do not report some symptoms to their physician, such as fatigue, and their impact on HRQoL and emotional wellbeing, because they perceive them as unrelated to their ITP or may feel intimidated by their physicians and not comfortable expressing their feelings or do not think their physician has enough time to discuss this when the patients are primarily concerned about bleeding, medication choice, or dosing. Regardless of the explanation, there is a need for physicians of all levels of experience to explore with their patients the emotional burden of ITP and to collaboratively seek solutions to address this despite the potentially limited time per visit. The findings from this survey on the impact of ITP on mental health resonate with those of a cohort study conducted in Denmark, which showed that individuals with ITP were 3.6‐fold more likely to have a mental health event than the general population in the first year after diagnosis [18].

### 4.2 Treatment Goals and Landscape

Nearly all patients (97%) reported having been on management for ITP at any time. In I‐WISh 1.0, corticosteroids were commonly prescribed for first and second relapse [12], but the most common second‐line treatments reported by physicians in I‐WISh 2.0 were TPO‐RAs. This reflects the 2019 American Society of Hematology (ASH) guideline and international consensus updates made available between the periods of the two surveys, as well as the influence of COVID‐19 in reducing steroid use in ITP [10, 25, 26]. However, at the time of the survey, most patients reported that they were receiving treatment: 24% with corticosteroids and 27% with TPO‐RAs. While reported corticosteroid use has reduced since I‐WISh 1.0, further reductions could still be achieved, especially with disease‐modifying therapy administered soon after diagnosis, instead of waiting for chronic ITP to develop [27, 28]. Furthermore, 82% of patients felt that sustained remission or a “cure” was important in terms of treatment preferences, with minimal side effects also being important. Although physicians and patients were generally satisfied with current therapeutic options for ITP, neither patients nor physicians felt the options were perfect.

Just over half of physicians were satisfied with current treatment options and approximately one‐third were neutral. Although patients were largely satisfied with their treatment, they were concerned about the short‐ and long‐term side effects and needing to be on treatment indefinitely. Almost one‐third of patients (31%) reported no change in symptoms compared with diagnosis. Similarly, although almost three‐quarters of patients generally felt that their current treatment was helping, many patients reported a significant psychological burden attached to their current ITP medication, including worrying about aspects such as immunosuppression and the emotional toll.

Although ranked differently, patients and physicians generally aligned on key treatment goals, consistent with those reported in I‐WISh 1.0 [12]. Consistent with the International Consensus Report on the management of ITP [10], key aims specified by physicians were to reduce bleeding, increase platelet count, and improve QoL. However, patients were more likely than physicians to report nonbleeding symptoms, such as fatigue and anxiety around unstable platelet counts. Most patients felt that not being on lifelong treatment (66%) and achieving sustained remission or a cure (82%) were important factors affecting treatment preference and decisions. Most physicians (71%) believed their patients would be willing to pause treatment following sustained response. Only 33% of patients reported having ever paused treatment (possibly reflecting a misunderstanding of the meaning of paused) and, at survey completion, 32% reported that they were not in sustained remission, i.e., did not have a stable platelet count. Of the patients reporting that they were in sustained remission, the reported duration of clinical remission (i.e., no bleeding and not receiving treatment) was ≤ 6 months in 51% and ≥ 12 months in only 33%. These results suggest that some of the current treatments do not achieve sustained remission following discontinuation and further studies are needed to understand the duration of remission following treatment discontinuation. While the ASH 2019 guideline defines remission as platelet count > 100 × 10^9^/L at 12 months [25], a specific definition was not applied to patient‐reported sustained remission in I‐WISh 2.0.

Although 73% of physicians consider patient views when making treatment decisions, I‐WISh 2.0 data suggest some discrepancies between patient and physician perspectives: 35% of patients worried they felt worse than their doctor thought they did, 28% agreed that they would be on a different treatment if it was their decision, and 24% indicated that they did not wish to receive treatment, but did so at their physicians' recommendation. However, these results should be interpreted with caution and provide guidance only, as patient preconceptions about treatments and the context and exact clinical scenarios surrounding treatment decisions in I‐WISh 2.0 are not known. When discussing treatment options, preventing immunosuppression was important for patients whereas this was a lesser priority for physicians. Patient concerns related to immunosuppression may reflect the increased fear as well as both awareness and misunderstanding of this term following the COVID‐19 pandemic, with common overestimation of its risk. Patient–physician communication should be further encouraged to educate on this topic, including that there are many degrees and forms of immunosuppression, as well as the redundancy of the immune system. Thus, there is usually limited predisposition to infectious disease, and numerous approaches to mitigate infection risk are available.

## 5 Limitations

Recruitment was lower than initial estimates based on I‐WISh 1.0, partially attributable to the COVID‐19 pandemic. There was no control group in I‐WISh 2.0, making it difficult to determine whether the patient insights were related to their ITP and its treatment or other unrelated factors, especially for fatigue. The online questionnaire and recruitment methods used meant it was not possible to identify how many patients had opened and started but not completed the survey, and the proportion of patients recruited online is not known. There was potential for bias since patients who had more severe symptoms and/or were more verbal may have been more motivated to participate and report impaired HRQoL than others. It is also likely that physicians more attuned to the impact of ITP on patients may have been more likely to participate in I‐WISh 2.0. Furthermore, details of ITP diagnosis and clinical information (e.g., platelet counts, symptoms) provided by patients were not requested; the information provided is based on patient perceptions (e.g., sustained remission is based on patient understanding of how well their disease is controlled rather than a measured platelet count response). Physicians' responses may have been influenced by knowledge of I‐WISh 1.0 results, but results were hopefully true to their practice.

The patients that physicians were describing when completing I‐WISh 2.0 are a separate population from the patients who completed the survey (because recruitment of the two groups was conducted independently). This may contribute to some of the different perceptions reported, although overall they were well aligned. It is important to recognize that physicians were selected for being familiar with ITP, more so than the majority of hematologists and hemato‐oncologists. Finally, because of the substantial accumulation of data, topics such as the impact of pregnancy, COVID‐19, and differences among countries had limited exploration. Despite these limitations, I‐WISh 2.0 provides the largest insight into characterizing patient and physician experiences with ITP since I‐WISh 1.0, building on previous findings.

## 6 Conclusions

ITP and its treatments impose substantial burdens on many patients. Bleeding, platelet counts, and treatments may be the most obvious, but the multifaceted effects on patient HRQoL, especially fatigue, are perhaps equally important. This includes effects on patients' day‐to‐day functioning, mental health, energy level, and emotional wellbeing. Patients and physicians were largely aligned on symptoms and treatment goals, with minor differences. Physicians were sensitive to the emotional burden of ITP on daily life, although, not surprisingly, patients were more likely than physicians to report nonbleeding symptoms, such as fatigue, depression, and anxiety around unstable platelet counts and long‐term treatment, as being important to them. Most patients and around half of physicians were generally satisfied with treatment for ITP, citing the ability to achieve their current treatment goals and, for physicians, the wide array of available treatments. Approximately two‐thirds of patients reported being in clinical remission; the majority of patients felt that a treatment offering sustained off‐treatment remission or a “cure” was important, ideally without side effects. Nonetheless, most patients, despite apparent willingness from most physicians, reported never pausing ITP treatment, although it is acknowledged that there may have been instances of reduced treatment dosing with falling platelet counts that prevented actually pausing treatment. Findings from I‐WISh 2.0 underscore the ongoing complex challenges of disease management and the importance of aligning treatment outcomes not only with patient expectations but also HRQoL outcomes. The results suggest that more attention should be paid to improving not only platelet counts, but also the HRQoL of patients. These considerations should be integrated into research, support programs and services in the future.

## Funding

Medical writing assistance was funded by Novartis Pharma AG in accordance with Good Publication Practice (GPP 2022) guidelines and funding was provided by Novartis Pharma AG to Adelphi Real World for the survey design, data collection, and data analysis.

## Ethics Statement

The study protocol and all survey materials were reviewed and approved by the Western Institutional Review Board.

## Consent

All survey participants provided consent.

## Conflicts of Interest

N.C. reports grants or contracts from Novartis and Rigel; consulting fees from Amgen, Grifols, Novartis, and Sobi; payment or honoraria for lectures, presentations, speakers' bureaus, manuscript writing, or educational events from Amgen, Grifols, Novartis, and Sobi; participation on a Data Safety Monitoring Board or Advisory Board for Sanofi, Novartis, Sobi, Grifols, Rigel, and Argenx; and is supported in part by the Imperial College National Institute for Health and Care Research Biomedical research Centre. J.B. reports consulting fees from Sobi, Argenx, UCB, Janssen, Mara Bio, Casi Pharmaceuticals, Amgen, and RallyBio; and payment or honoraria for lectures, presentations, speakers' bureaus, manuscript writing, or educational events from UpToDate. W.G. reports grants or contracts: Sobi, Grifols, and Sanofi; consulting fees from Sobi, Grifols, Sanofi, Takeda, Novartis, and Argenx; and payment or honoraria for lectures, presentations, speakers' bureaus, manuscript writing, or educational events from Sobi, Grifols, Sanofi, Takeda, Novartis, and Argenx. D.P. reports consulting fees from Climb Bio, Takeda, Nuvig, Dexel, and Sobi (received by his business, Herodotus Ltd.); payment or honoraria for lectures, presentations, speakers' bureaus, manuscript writing, or educational events from Amgen, Novartis, Sobi, and Grifols (received by his business, Herodotus Ltd.); support for attending meetings and/or travel from Novartis (received by his business, Herodotus Ltd.); participation on a Data Safety Monitoring Board or Advisory Board for Novartis and Climb Bio (received by his business, Herodotus Ltd.); and stock or stock options from GSK. Y.T. reports consulting fees from Novartis, Argenx, Sobi, Sanofi, Kissei, Asahi Kasei, and Takeda; payment or honoraria for lectures, presentations, speakers' bureaus, manuscript writing, or educational events from Novartis, Kyowa‐Kirin, Sobi, Sysmex, Kissei, and Asahi Kasei; participation on a Data Safety Monitoring Board or Advisory Board for Novartis; and support for the present manuscript from Novartis. D.M.A. reports support for the present manuscript from Novartis; royalties or licenses from UpToDate; consulting fees from Amgen, Medison, Sobi, Argenx, and Sanofi; participation on a Data Safety Monitoring Board or Advisory Board for Takeda; and being a medical advisor (unpaid) for the Platelet Disorders Support Association (PDSA). C.S. reports consulting fees from Grifols, Amgen, Novartis, and Sobi; and payment or honoraria for lectures, presentations, speakers' bureaus, manuscript writing, or educational events from Sobi, Novartis, and Amgen. F.Z. reports grants or contracts from Novartis, Amgen, and Sobi (no personal compensation received); consulting fees from Novartis, Amgen, Argenx, Grifols, BeiGene, Lilly, Janssen, Takeda, Gilead, AbbVie, Sanofi, AstraZeneca, and Incyte; payment or honoraria for lectures, presentations, speakers' bureaus, manuscript writing, or educational events from Novartis, Amgen, Argenx, Grifols, Sobi, AbbVie, Gilead, Takeda, Lilly, BeOne, and Janssen; support for attending meetings and/or travel from Jazz, Janssen, BeOne, Grifols, AbbVie, and Amgen; and participation on a Data Safety Monitoring Board or Advisory Board for Novartis, Amgen, Argenx, Grifols, BeOne, Lilly, Janssen, Takeda, Gilead, AbbVie, and Sanofi. B.L. reports consulting fees from Novartis Pharma AG; and payment or honoraria for lectures, presentations, speakers' bureaus, manuscript writing, or educational events from Open Health Communications LLP, Swedish Orphan Biovitrium AB, UCB Biopharma S.r.l., GCO (Global Conference Organizers) B.V., ELLEVENTI S.r.l., Genzyme Corporation, and Argenx BV. M.M. reports consulting fees from Novartis and Sobi (payment to organization); support for attending meetings and/or travel from Novartis and Sobi (payment to organization); and participation on a Data Safety Monitoring Board or Advisory Board for Sobi, Novartis, Amgen, Argenx, Sanofi, Takeda, and Alpine (payment to organization). J.D. reports being a Board of Directors member (treasurer) for the Network of Rare Blood Disorder Organizations in Canada (volunteer); being on the Board of Directors for the Canadian Hemophilia Society (volunteer); being Chair of the Canadian Association of Genetic Counselors research grant subcommittee (volunteer); and being a patient advisor representing PDSA, which received grants and consultancy fees from Novartis, Sobi, Sanofi, Amgen, and Argenx (no personal compensation received). D.B. reports being a Management Board member (secretary) for the International ITP Alliance (volunteer); and being Chief Executive Officer of ITP Australia and New Zealand (ITP Australia Ltd.; part‐time employee and volunteer). O.R.‐H. reports being an employee of Adelphi Real World. M.V. reports stock or stock options from Novartis Pharma AG. S.F. reports being an employee of Novartis; and stock or stock options from Novartis Pharma AG. C.K. reports being Board Chair of the International ITP Alliance (volunteer); and being a patient advisor representing PDSA for Novartis (no personal compensation received). M.H. and M.W. have no conflicts to declare.

## Supporting information


**Table S1:** Change in platelet count from ITP diagnosis to most recent platelet count test (*N* = 850).
**Table S2:** Change in number of ITP symptoms at the time of survey completion compared with diagnosis.
**Table S3:** Proportion of patients reporting a high impact of ITP on daily activities and family/social life, by (a) disease phase and (b) platelet count.
**Table S4:** Avoidance of physical activity, by age, disease phase, platelet count, and fatigue.
**Table S5:** Proportion of patients reporting a high impact of ITP on emotional wellbeing, by (a) disease phase and (b) platelet count.
**Figure S1:** (a) Physician perception of patients' willingness to pause treatment and (b) impact of limiting time on treatment/not being on treatment on patients' treatment preference.

## Data Availability

Novartis is committed to sharing with qualified external researchers access to patient‐level data and supporting clinical documents from eligible studies. These requests are reviewed and approved by an independent review panel on the basis of scientific merit. All data provided are anonymized to respect the privacy of patients who have participated in the trial, in line with applicable laws and regulations. This trial data availability is according to the criteria and process described on www.clinicalstudydatarequest.com.
