# Supplementary material for: Exploring the Burden on Patients Living With and Receiving Treatment for Immune Thrombocytopenia (ITP): Patient and Physician Perceptions From the ITP World Impact Survey (I‐WISh) 2.0
Source: Am J Hematol. 2026 Jun 3;101(8):1954–68. doi: 10.1002/ajh.70379 (PMC13331645; doi:10.1002/ajh.70379)
Supplement: Supplementary file 1 — Table S1: Change in platelet count from ITP diagnosis to most recent platelet count test (N = 850). Table S2: Change in number of ITP symptoms at the time of survey completion compared with diagnosis. Table S3: Proportion of patients reporting a high impact of ITP on daily activities and family/social life, by (a) disease phase and (b) platelet count. Table S4: Avoidance of physical activity, by age, disease phase, platelet count, and fatigue. Table S5: Proportion of patients reporting a high impact of ITP on emotional wellbeing, by (a) disease phase and (b) platelet count. Figure S1: (a) Physician perception of patients' willingness to pause treatment and (b) impact of limiting time on treatment/not being on treatment on patients' treatment preference. [file AJH-101-1954-s001.pdf]

Exploring the Burden on Patients Living With and Receiving Treatment for Immune Thrombocytopenia (ITP): Patient and Physician Perceptions from the ITP World Impact Survey (I WISh) 2.0

Cooper N, Bussel J, Ghanima W et al.

Supplemental Data

Rationale

The aim of I-WISh (ITP World Impact Survey) 2.0 was to follow-up and expand on the I-WISh 1.0 survey, a patient and physician survey in ITP with an objective to understand the overall burden of immune thrombocytopenia (ITP) on patients, as well as assessing patient and physician perceptions on the current management of the disease [1, 2].

Addition of new countries to I-WISh 2.0 allowed for expansion of the patient/physician sample, and increased global representativeness, adding to the value of the original study. Additional objectives allowed for a more in-depth review of the burden of fatigue and emotional impact of living with ITP.

Methods

Participants

- Physicians – Hematologists or hemato-oncologists who actively managed patients with ITP and had a minimum caseload of three ITP patients in the 12 months preceding the survey
- Patients – Patients aged >18 years with a diagnosis of ITP willing and able to provide consent and complete the survey independently

Study Design

This was an exploratory, cross-sectional study that administered questionnaires to both physicians who treated patients with ITP and patients diagnosed with ITP.

Data collected in the online physician questionnaire included but were not limited to:

- Demographics
  - Specialty
  - Care setting
  - Length of time practicing medicine
  - Year of qualification
  - Length of time treating ITP patients
- Symptoms
  - Symptoms most frequently heard about from ITP patients
  - Most common symptoms at time of ITP diagnosis
  - Common locations of hematoma
  - Impact of symptoms on quality of life (QoL)
  - Impact of ITP-related fatigue on QoL

- Impact of fatigue
  - Perception of educating patients on fatigue as symptom of ITP
  - Perception of patient’s fatigue as a result of ITP
  - Estimated proportion of ITP patients who experience psychological symptoms
  - Method and frequency in which fatigue is assessed in ITP patients
  - Perception of association between patient’s platelet count and level of fatigue
  - Perception of frequency to which ITP patients sleep during the day
  - Perception of reasons for patient’s fatigue
- Impact on daily life
  - Perception of how ITP impacts patient’s ability to work
  - Perception of the extent to which ITP impacts patient’s career
  - Perception of impact on patient’s income due to ITP
  - Perception of impact on patient’s ability to concentrate on daily tasks
  - Perception of impact on patient’s physical activity levels
  - Perception of impact on patient’s sex life
  - Perception of impact on patient’s ability to travel
- Disease management and treatment approaches
  - Treatment utilization for ITP at different lines
  - Treatment goals
  - Satisfaction with current treatment options
  - Perceptions of patient’s understanding of treatment goals
  - Reasons to change patient’s drug therapy
  - Perception of corticosteroid impact on patients’ QoL
  - Estimated proportion of ITP patients to undergo platelet destruction
  - Reasons to pause or discontinue patient’s treatment
  - Perception of negative impacts on patient’s fatigue
  - Dietary changes due to ITP treatment
  - Perception of patient’s burden in diet change
- Emotional impact
  - Perception of patient’s overall well-being because of ITP
  - Perception of impact on family and friends
  - Perception of patient’s worry due to ITP
  - Estimated proportion of ITP patients prescribed anxiety/depression medication
  - Perception of patient’s experiences/feelings with ITP
  - Estimated proportion of ITP patients receiving professional support
- Impact on pregnancy
  - Impact on emotional wellbeing during pregnancy
  - Treatment of patients with ITP during pregnancy
- Impact of COVID-19 on ITP
  - Impact on patient management for ITP

- Change in prescription of medication

The main components of the patient questionnaire included, but were not limited to:

- Demographics
  - Gender
  - Length of time since ITP diagnosis
  - Initial suspicion of ITP
  - Current enrollment in an ITP-related clinical trial study
  - Concomitant conditions
- Symptoms
  - Current state of health
  - Symptomatology currently
  - Symptomatology at ITP diagnosis
  - Severity of symptoms currently
  - Platelet count (at diagnosis, at most recent test)
  - Stability of platelet counts currently
  - Number of platelet count tests in past 12 months
- Impact of fatigue
  - Modified Fatigue Impact Scale – 5-item version (MFIS-5)
  - Assessment of fatigue at routine appointments for ITP
  - Explanation of fatigue as a symptom
  - Reasons for fatigue
  - Frequency of sleep during the day
  - Hours of sleep (daytime and night-time)
  - Sleep pattern/quality
- Impact on daily life
  - ITP Life Quality Index (ILQI)
  - Work Productivity and Activity Impairment Questionnaire: Specific Health Problem V2.0 (WPAI:SHP)
  - Current employment status
  - Kind of work are/were doing
  - Current employer awareness of ITP
  - Current total household income
  - ITP impact on career
  - Health insurance coverage
  - Help/support required
  - ITP impact on physical activity
- Treatment for ITP
  - Management approaches ever received for ITP
  - Management approach currently receiving for ITP
  - Time since last treatment
  - Other prescribed treatments for other conditions
  - Platelet destruction test

    - Sustained remission
    - Top treatment goals
    - Healthcare professional awareness of treatment goals
    - Pause in treatment
    - Dose reduction
- Emotional impact of ITP
    - Impact on emotional wellbeing
    - Impact of starting treatment on emotional wellbeing
    - Medication for depression/anxiety
    - Professional support
    - Support from family/friends
    - Patient Health Questionnaire-9 (PHQ-9)
    - Impact on partner/spouse/child/parents/sibling/other family
- Impact on pregnancy
    - Positive/negative experience due to ITP
    - Experience during current/most recent pregnancy
    - Impact on emotional well-being during pregnancy
- Impact of COVID-19 on ITP
    - COVID-19 confirmation with test
    - Symptoms of COVID-19
    - COVID-19 effect on ITP treatment
    - COVID-19 effect on platelet count

Some responses were classified on 1–7 Likert scales (5–7 indicating a high impact/agreement/severity/importance or great deal). The PHQ-9 (scale of 0–27) and MFIS (0=never, 5=almost always) assessed depression and fatigue, respectively. Patients also completed the WPAI:SHP (0% indicating no impairment and 100% maximum impairment) to assess the impact of ITP on work and daily activities.

Survey materials were developed in English and then translated into the appropriate language (Simplified Chinese, Arabic, Hebrew, French, German, Italian, Japanese, Norwegian, Spanish, South Korean, Hindi, and Telugu) by an accredited translation agency. Translations were verified by local proof-readers in each country, who approved the accuracy of the translations.

Data collection was conducted by Adelphi Real World (ARW) through local fieldwork partners in each country. Data collection was initially soft launched in the UK and USA. Data captured during the soft launch phase were reviewed to ensure the programmed surveys functioned correctly. Following review and sign-off of the soft launch data, the study was rolled out to the full sample.

Physician survey: Prior to data collection, eligible physicians were identified and contacted by the local fieldwork partner in each country. Local fieldwork partners provided physicians with information about the online survey and instructions about completion. Providing a link and using fieldwork partners ensured that neither ARW nor Novartis were aware of

the identity of participating physicians. Each survey was identified with a survey number to ensure anonymity.

Patient survey: Physicians or patient advocacy groups (PAGs) approached patients diagnosed with ITP and invited them to complete the survey. Physicians were identified by ARW to facilitate the recruitment of ITP patients. Local fieldwork partners provided physicians with an overview of the survey. Physicians identified eligible patients during routine consultations and provided them with information about the study and how to complete the questionnaire. Patients were also recruited through PAGs. Patients who were willing to participate were directed to a link if they were accessing the online survey. Each survey was identified with a survey number to ensure anonymity. The use of fieldwork partners, PAGs, and providing a link to complete the surveys ensured that neither ARW nor Novartis were aware of the identity of any of the patient participants. To ensure the participants were those with a diagnosis of ITP, all participants had to confirm their diagnosis during a screening phase prior to completing the survey to establish their eligibility.

Data Management

Data were transferred to a single electronic database. Analyses were conducted using Stata Statistical Software Version 16.0 or later (Stata statistical software: Release 16. College Station, TX: StataCorp LP, 2015).

Participants with missing data were removed from all data summaries associated with that variable but remained eligible for inclusion in other data summaries.

Data Analysis

As there were no pre-specified hypotheses in this exploratory study, no statistical analyses were undertaken. Data were summarized narratively using descriptive statistics. For categorical variables, the percentage of responses, total number of respondents who gave that answer (*n*) and total number of respondents who completed the question (*N*) are shown. For numeric variables, the respondent base, mean, standard deviation, median, quartiles and range (minimum and maximum values) are reported.

Subgroup definitions

Subgroup	Question subgroup was derived from and responses considered for subgroup analyses
Patient fatigue	Which of the following symptoms, if any, did/do you have in the past month?<br>• Patient indicated fatigue<br>• Patient did not indicate fatigue
Patient's current health	How would you describe your current health?<br>• Low levels of current health (score of 1–3)<br>• Moderate levels of current health (score of 4–5)<br>• High levels of current health (score of 6–7)
Patient platelet count at ITP diagnosis	What was/is your platelet count at the time of ITP diagnosis?<br>• <10 ($\times10^9$/L)<br>• 10–19 ($\times10^9$/L)<br>• 20–29 ($\times10^9$/L)<br>• 30–49 ($\times10^9$/L)<br>• 50–99 ($\times10^9$/L)<br>• ≥100 ($\times10^9$/L)<br>• Don't know
Patient platelet count at recent test	What was/is your platelet count at the most recent test?<br>• <10 ($\times10^9$/L)<br>• 10–19 ($\times10^9$/L)<br>• 20–29 ($\times10^9$/L)<br>• 30–49 ($\times10^9$/L)<br>• 50–99 ($\times10^9$/L)<br>• ≥100 ($\times10^9$/L)<br>• Don't know
Patient-reported stable platelet count	Are you in a period of sustained remission (i.e., your platelet count is stable) in your ITP?<br>• Yes<br>• No<br>• Don't know
Duration of patient-reported no bleeding and no treatment	How long have you been in clinical remission (i.e., no bleeding and no treatment)?<br>• 0–3 months<br>• 4–6 months<br>• 7–12 months<br>• >12 months
Patient dose reduction	Before pausing treatment, was the dose reduced?<br>• Yes<br>• No<br>• Don't know

Patient treatment pause	Subgroup derived from question restarting it a later point (please only consider treatment pause that was advised by your doctor)?<br>• Yes<br>• No<br>• Don’t know
Phase of disease	How long since you were diagnosed with ITP?<br>• Newly diagnosed (0–3 months)<br>• Persistent (4–12 months)<br>• Chronic (>12 months)

Abbreviation: ITP, immune thrombocytopenia.

Subgroup analyses

The proportion of patients and/or physicians reporting a high impact of ITP on daily activities, family/social life and emotional wellbeing was also assessed according to disease phase and platelet count. Avoidance of physical activity was assessed according to age, disease phase, platelet count and reporting of fatigue.

Results

Prevalence of ITP Symptoms at Diagnosis

The prevalence of some symptoms was similar at diagnosis and during the month prior to survey completion: 54% (549/1018) of patients experienced fatigue at diagnosis compared with 48% (490/1018) in the month prior to survey completion; and 28% (286/1018) reported anxiety around their platelet count at diagnosis and 28% (284/1018) experienced this symptom in the month before survey completion. Interestingly, patients reported depression, joint pain and an increased number of moderate to severe headaches/migraines in their top 10 symptoms in the month prior to survey completion but not at diagnosis. The most common symptoms reported by physicians were similar at diagnosis and in the month prior to survey completion, although more physicians reported patients mentioning anxiety around platelet count in the month prior to survey completion (19%, 83/431) than at diagnosis (11%, 49/431).

Physician perception of impact of ITP on daily life

Physicians estimated that roughly half or more of their patients (includes physicians who answered ‘about 50%’, ‘most’, or ‘all’ patients) are less alert (41%, 172/422) and have trouble maintaining physical (64%, 276/428) or mental effort (56%, 238/427) for long periods as a result of their ITP. Physicians (79%, 342/431) agreed that, in relation to traveling, patients have concerns around an increased risk of bleeding; 50% of patients agreed (481/955) that when thinking about travel plans they worry about an increased risk of bleeding. 43% of patients (401/928) agreed that it is inconvenient to consult about their platelet count before traveling, whereas 71% (306/431) of physicians thought that their patients find this burdensome.

Impact of ITP on sleep

Most patients (57%, 491/855) believed (5–7 on a Likert scale) that their sleep quality would be better and 56% (487/866) believed their sleep pattern would be more stable if they did not have ITP. 58% of physicians (243/422) estimated that about 50% or more of their patients (i.e., physicians who answered, ‘about 50%’, ‘most’, or ‘all’ patients) have a poor quality of sleep and 57% (240/419) of physicians estimated that about 50% or more patients have an unstable sleep pattern.

Supplementary Table S1. Change in platelet count from ITP diagnosis to most recent platelet count test (*N* = 850). Blue shaded areas show number of patients with no change in platelet count

		Platelet count at most recent test, *n*						
		<10×10^9/L	10–19×10^9/L	20–29×10^9/L	30–49×10^9/L	50–99×10^9/L	100–199×10^9/L	≥200×10^9/L
		34	33	63	122	245	235	118
**Platelet count at diagnosis, *n***								
<10×10^9/L	304	23	15	23	29	51	94	69
10–19×10^9/L	162	7	7	14	27	51	37	19
20–29×10^9/L	139	2	7	11	26	55	30	8
30–49×10^9/L	131	1	3	7	24	48	36	12
50–99×10^9/L	95	1	0	5	14	38	31	6
100–199×10^9/L	15	0	1	2	2	1	6	3
≥200×10^9/L	4	0	0	1	0	1	1	1

Abbreviation: ITP, immune thrombocytopenia.

Supplementary Table S2. Change in number of ITP symptoms[a] at the time of survey completion compared with diagnosis

	n	%
**Change in number of ITP symptoms (time of survey completion compared with diagnosis)**	1006	
Increase	175	17
No change	170	17
Decrease	661	66
**Percentage change in number of ITP symptoms (time of survey completion compared with diagnosis)**	957	
>0–<25% decrease	52	5
25–50% decrease	140	15
>50% decrease	469	49
No change	137	14
>0–<25% increase	16	2
25–50% increase	28	3
>50% increase	115	12

Abbreviation: ITP, immune thrombocytopenia.

[a]Symptoms enquired about included bleeding, headaches/migraines, dizziness, depression, anxiety, gastrointestinal symptoms (i.e., reduced appetite, stomach pain and indigestion), joint pain, and chest pain.

Supplementary Table S3. Proportion of patients reporting a high impact of ITP on daily activities and family/social life, by (a) disease phase and (b) platelet count

(a)

**Disease phase**	**High impact reported by patients, % (*n/N*)**	
	**Daily activities**	**Family/social life**
All (*n* = 1018)	33 (320/983)	35 (348/984)
Newly diagnosed (*n* = 22)	41 (9/22)	38 (8/21)
Persistent (*n* = 127)	40 (49/121)	43 (52/122)
Chronic (*n* = 869)	31 (262/840)	34 (288/841)

(b)

**Platelet count (×10^9/L)**	**High impact reported by patients, % (*n/N*)**	
	**Daily activities**	**Family/social life**
All (*n* = 1018)	33 (320/983)	35 (348/984)
<100 (*n* = 555)	38 (203/541)	40 (219/543)
<10 (*n* = 36)	41 (14/34)	54 (19/35)
10–19 (*n* = 41)	33 (13/39)	42 (16/38)
20–29 (*n* = 70)	47 (31/66)	41 (28/68)
30–49 (*n* = 139)	49 (68/139)	52 (72/139)
50–99 (*n* = 269)	29 (77/263)	32 (84/263)
≥100 (*n* = 392)	27 (103/375)	29 (110/376)
100–199 (*n* = 263)	27 (69/255)	29 (74/254)
≥200 (*n* = 129)	28 (34/121)	30 (36/121)

Abbreviations: ITP, immune thrombocytopenia.

Supplementary Table S4. Avoidance of physical activity, by age, disease phase, platelet count, and fatigue

	Avoiding moderate physical activity, % (*n*/*N*)	Avoiding intense physical activity, % (*n*/*N*)
**Overall population** (*n* = 1018)	32 (312/976)	64 (625/970)
**Age (years)**		
18–40 (*n* = 380)	35 (131/373)	65 (241/371)
41–60 (*n* = 415)	31 (122/398)	64 (253/396)
≥61 (*n* = 233)	29 (59/205)	65 (131/203)
**Disease phase**		
Newly diagnosed (*n* = 22)	38 (8/21)	59 (13/22)
Persistent (*n* = 127)	42 (50/120)	67 (80/119)
Chronic (*n* = 869)	30 (254/835)	64 (532/829)
**Platelet count (×10^9/L)**		
<100 (*n* = 555)	36 (191/534)	70 (371/531)
≥100 (*n* = 392)	29 (108/378)	60 (224/376)
**Fatigue**		
Yes (*n* = 490)	40 (192/478)	76 (363/478)
No (*n* = 528)	24 (120/498)	53 (262/492)

Supplementary Table S5. Proportion of patients reporting a high impact of ITP on emotional wellbeing, by (a) disease phase and (b) platelet count

(a)

**Disease phase**	**Patients reporting high impact on emotional wellbeing, % (*n*/*N*)**
All (*n* = 1018)	54 (554/1017)
Newly diagnosed (*n* = 22)	59 (13/22)
Persistent (*n* = 127)	63 (80/127)
Chronic (*n* = 869)	53 (461/868)

(b)

**Platelet count (×10^9/L)**	**Patients reporting high impact on emotional wellbeing, % (*n*/*N*)**
All (*n* = 1018)	54 (554/1017)
<10 (*n* = 36)	64 (23/36)
10–19 (*n* = 41)	54 (22/41)
20–29 (*n* = 70)	63 (44/70)
30–49 (*n* = 139)	69 (96/139)
50–99 (*n* = 269)	52 (140/269)
100–199 (*n* = 263)	50 (131/263)
≥200 (*n* = 129)	52 (67/129)
<100 (*n* = 555)	59 (325/555)
≥100 (*n* = 392)	51 (198/392)

Abbreviations: ITP, immune thrombocytopenia.

Supplementary Figure S1. (a) Physician perception of patients' willingness to pause treatment and (b) impact of limiting time on treatment/not being on treatment on patients' treatment preference

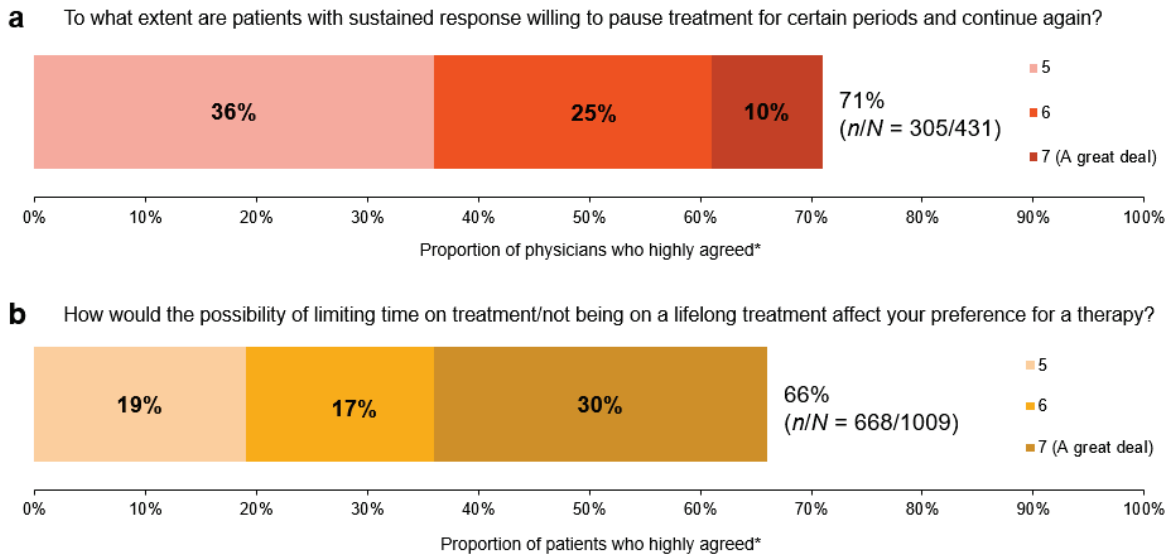

n represents the total number of participants who gave this answer and *N* denotes the total number of participants who completed the question.

*Highly agreed meant scoring 5–7 on a Likert scale.

References

1. N. Cooper, A. Kruse, C. Kruse, et al., "Immune Thrombocytopenia (ITP) World Impact Survey (I-WISh): Impact of ITP on Health-Related Quality of Life," *American Journal of Hematology* 96, no. 2 (2021): 199–207, https://doi.org/10.1002/ajh.26036.
2. N. Cooper, A. Kruse, C. Kruse, et al., "Immune Thrombocytopenia (ITP) World Impact Survey (iWISh): Patient and Physician Perceptions Of Diagnosis, Signs and Symptoms, and Treatment," *American Journal of Hematology* 96, no. 2 (2021): 188–198, https://doi.org/10.1002/ajh.26045.
